# A case of duodenal malignant lymphoma presenting as acute pancreatitis: systemic lupus erythematosus and immunosuppressive therapy as risk factors

**DOI:** 10.1007/s12328-018-0848-2

**Published:** 2018-03-15

**Authors:** Reiko Yamada, Takashi Sakuno, Hiroyuki Inoue, Hiroshi Miura, Toshifumi Takeuchi, Yasunori Shiono, Hiroaki Okuse, Misaki Nakamura, Masaki Katsurahara, Yasuhiko Hamada, Kyosuke Tanaka, Noriyuki Horiki, Yoshiyuki Takei

**Affiliations:** 1 Department of Gastroenterology and Hepatology, Mie University Hospital, Mie University Graduate School of Medicine, 2-174 Edobashi, Tsu, Mie 514-8507 Japan; 2 Department of Endoscopy, Mie University Graduate School of Medicine, Tsu, Japan

**Keywords:** Duodenal malignant lymphoma, Pancreatitis, Systemic lupus erythematosus (SLE), Tacrolimus, Immunosuppressive therapy-associated lymphoproliferative disorders

## Abstract

A 49-year-old man was admitted to our hospital with pancreatitis. He was diagnosed with systemic lupus erythematosus at 34 years of age and was being treated with oral tacrolimus (3 mg/day) and predonine (10 mg/day) for the past 15 months. The computed tomography (CT) scan showed the mass lesion had invaded the pancreatic head via thickening of the duodenal wall. Upper gastrointestinal endoscopy showed the all-round ulcerative lesion from the superior duodenal angle to the descending portion. Histological examination confirmed the diagnosis of diffuse large B cell lymphoma (DLBCL). Tacrolimus therapy was stopped due to the possibility of immunodeficiency-related lymphoproliferative disease; however, the lesion did not improve. Consequently, he was administered rituximab plus cyclophosphamide, doxorubicin, vincristine, and prednisone (R-CHOP). After six courses of R-CHOP therapy, a partial response was confirmed on CT. One month after the completion of chemotherapy, a gastrojejunal anastomosis was performed because of duodenal stenosis. He has since been well without recurrence. It was difficult to identify the risk factor for DLBCL; therefore, both the disease activity and immunosuppressive therapy should be taken into consideration as carrying a risk. In the present case, the symptom of pancreatitis enabled an early diagnosis of DLBCL.

## Introduction

Extra-nodal non-Hodgkin’s lymphoma (NHL) constitutes 30–40% of all cases of lymphoma [1]. Gastrointestinal tract lymphomas are the most common type of extra-nodal NHL, accounting for approximately 10% of NHL cases [2]. However, secondary involvement of the pancreas has been reported less frequently; only 0.2–2% of patients with lymphoma have pancreatic involvement at the time of diagnosis [3]. Moreover, there have been only two cases of secondary pancreatic involvement by lymphoma presenting as acute pancreatitis [3, 4]; these two cases described involvement from an adjacent peripancreatic lymphadenopathy and not from duodenal lymphoma as described in the present case.

Some studies have demonstrated that chronic inflammation might heighten the lymphoma risk in patients with chronic autoimmune diseases, such as systemic lupus erythematosus (SLE), compared to the general population [5]. On the contrary, a relationship between exposure to immunosuppressive therapy and lymphoma development has also been shown in these patients. Immunosuppressive therapy-associated lymphoproliferative disorders are known to occur following treatment with immunosuppressive agents, such as methotrexate, azathioprine, and infliximab [6].

The patient in this case report had no primary immunodeficiency and no history of human immunodeficiency virus infection or organ transplantation; however, he developed duodenal NHL after tacrolimus treatment for SLE. This case also had a unique clinical course with secondary pancreatic involvement of duodenal NHL presenting as acute pancreatitis.

## Case report

**Fig. 1:**
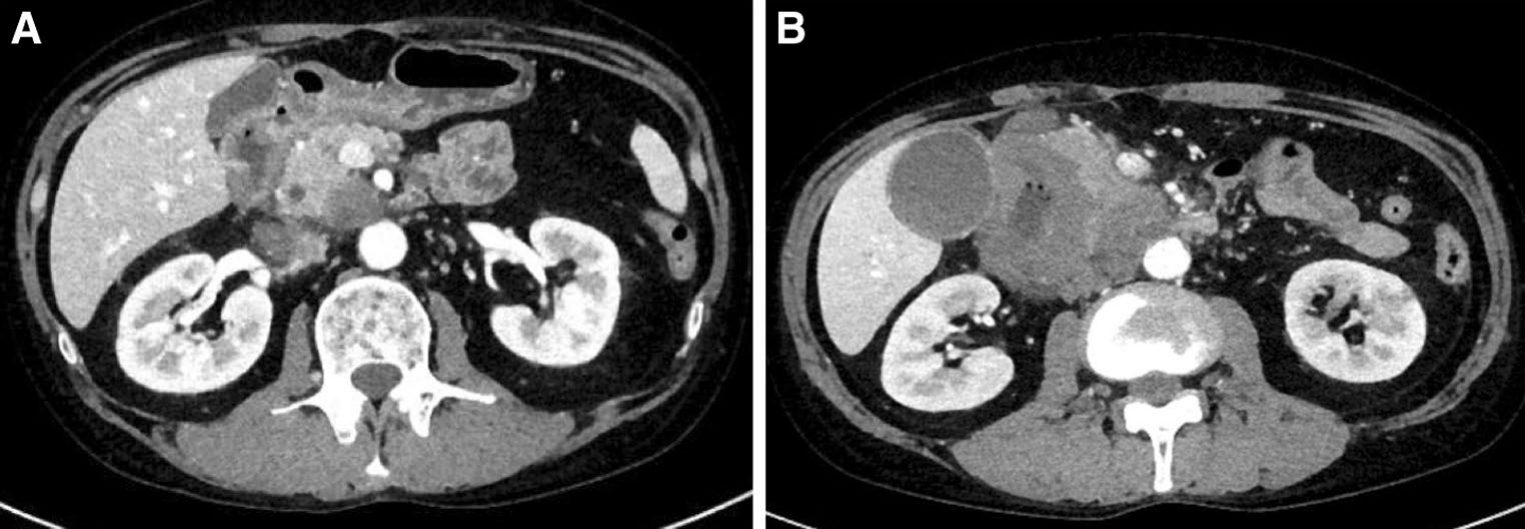
The CT scan showed pancreatic head swelling and slight peripancreatic fluid effusion (**a**). The enhanced CT scan showed an all-round wall thickening of the descending portion of the duodenum and the ill-defined mass lesion invading the pancreatic head (**b**)

A 49-year-old man presented to our hospital with severe, continuous epigastric pain. Initial laboratory tests showed increased amylase (AMY; 823 IU/L) and lipase (465 U/L) levels, as well as an increased white blood cell count (WBC; 11,160/μL) and C-reactive protein levels (CRP; 8.21 mg/dL). He was admitted with the diagnosis of mild pancreatitis based on the laboratory results and computed tomography (CT) findings of pancreatic head swelling and slight peripancreatic fluid effusion (Fig. 1a). There were no stones observed both in the bile duct or the gallbladder. The bedside index for severity in acute pancreatitis (BISAP) score was calculated to be 0. The patient was treated conservatively with fasting and fluid hydration.

The patient was diagnosed with SLE at 34 years of age and was being treated with an oral immunosuppressant (tacrolimus; 3 mg/day) in addition to a steroid (predonine; 10 mg/day) for the past 15 months. His history also included a laparoscopic ileostomy performed 2 months before admission due to an intractable rectal ulcer.

**Fig. 2:**
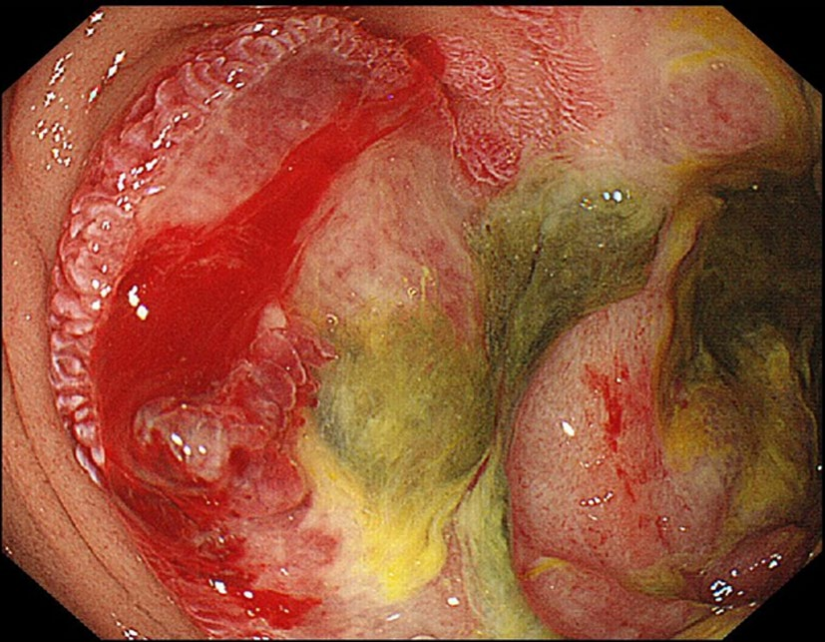
An upper gastrointestinal endoscopy showed the all-round ulcerative lesion over a large region from a superior duodenal angle (SDA) to the descending portion of duodenum. The well-demarcated lesion consisted of an ulcer with a regular elevated margin that had an auricle-like shape

**Fig. 3:**
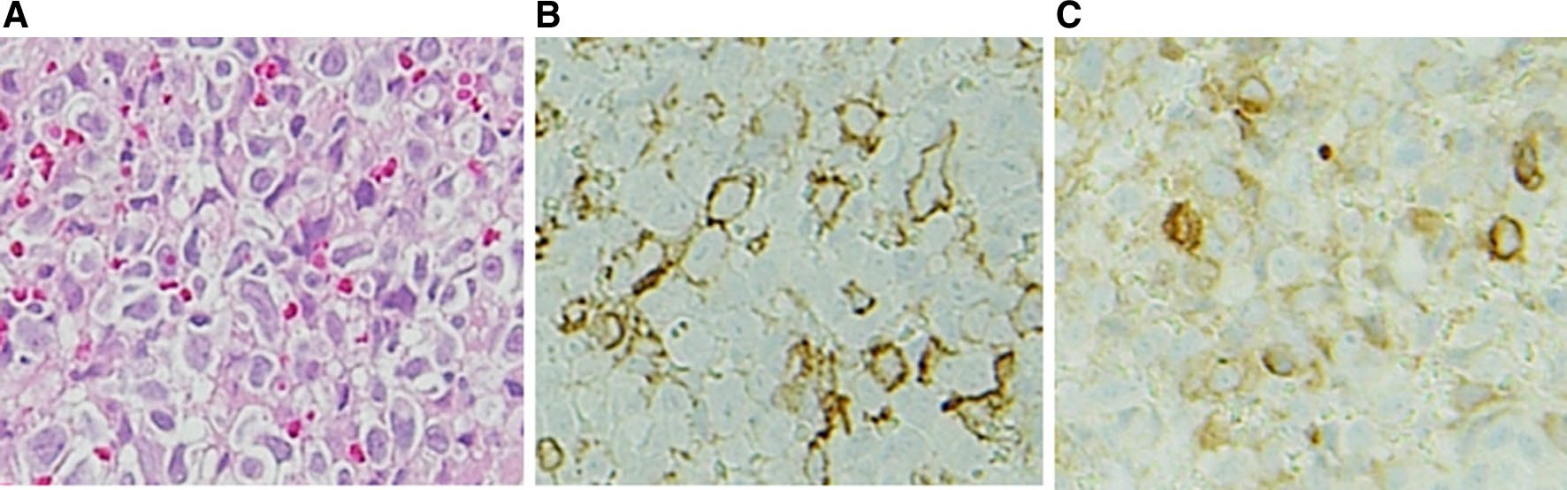
Histological examination of the biopsy specimens confirmed the diagnosis of diffuse large B-cell lymphoma (DLBCL) (**a**); immunochemical staining was positive for CD20 (**b**) and CD79a (**c**)

**Fig. 4:**
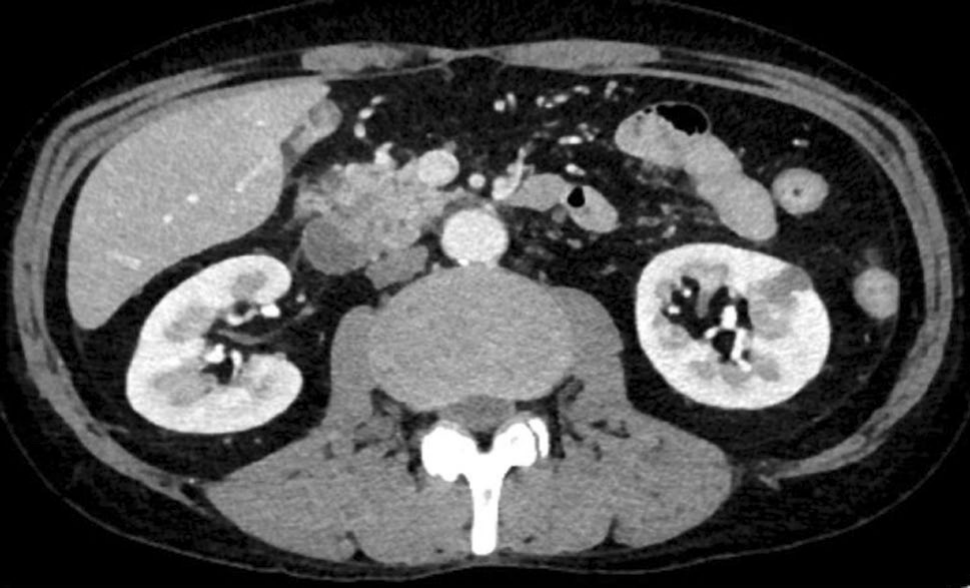
After six courses of R-CHOP therapy, a 95.3% lesion rate of reduction was confirmed on CT, which was judged as a partial response

**Fig. 5:**
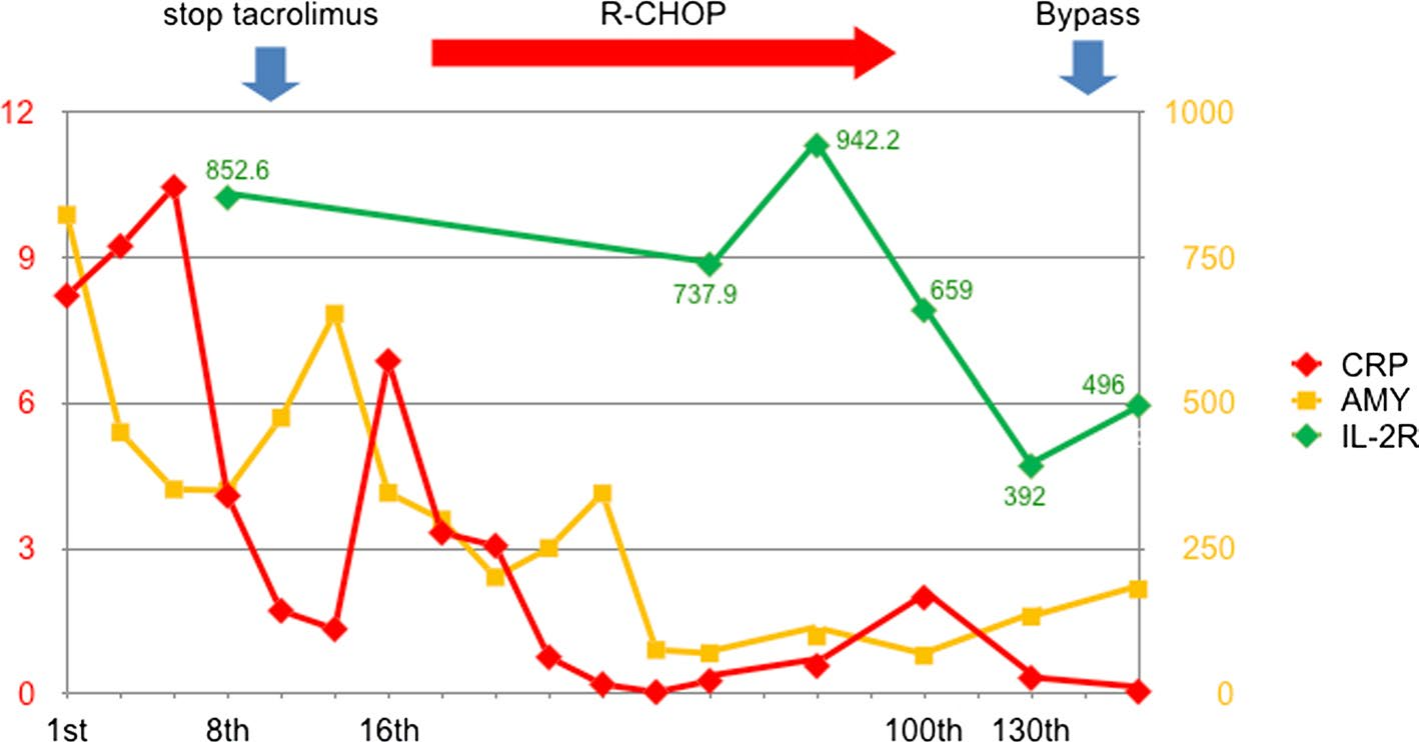
The chart shows that the initial laboratory tests indicated increased amylase (AMY; 823 IU/L) and C-reactive protein levels (CRP; 8.21 mg/dL). His abdominal pain was sustained on the day 8 after admission, although the inflammatory reaction and pancreatic enzyme levels were improving with fasting and infusion therapy: CRP = 4.09 mg/dL, and AMY = 350 IU/L. Tacrolimus was stopped on day 9, but the lesion continued to increase and his abdominal pain still persisted. Consequently, he was administered rituximab plus cyclophosphamide, doxorubicin, vincristine, and prednisone (R-CHOP) therapy from day 16. His symptoms improved and the laboratory data showed a good clinical course

The enhanced CT scan showed an all-round wall thickening of the descending portion of the duodenum. The ill-defined mass lesion had invaded the pancreatic head (Fig. 1b). An upper gastrointestinal endoscopy showed the all-round ulcerative lesion over a large region from the superior duodenal angle (SDA) to the descending portion of the duodenum. This well-demarcated lesion consisted of an ulcer with a regular elevated margin that had an auricle-like shape (Fig. 2). Histological examination of the biopsy specimens confirmed the diagnosis of diffuse large B-cell lymphoma (DLBCL); immunochemical staining was positive for CD20 and CD79a (Fig. 3a–c). He was negative for Epstein–Barr virus (EBV). His abdominal pain continued to persist on the day 8 after admission, although his inflammatory reaction and pancreatic enzyme levels were improving with fasting and infusion therapy (WBC = 6300/μL, CRP = 4.09 mg/dL, and AMY = 350 IU/L). Tacrolimus therapy was stopped on day 9 in view of the possibility of “immunodeficiency-related lymphoproliferative disease.” In spite of tacrolimus withdrawal, the lesion continued to increase and his abdominal pain remained. Consequently, he started rituximab plus cyclophosphamide, doxorubicin, vincristine, and prednisone (R-CHOP) therapy from day 16 after admission. After six courses of R-CHOP therapy, a 95.3% lesion reduction rate was confirmed on CT, which was judged as a partial response (Fig. 4). The symptoms had improved and the laboratory data showed a good clinical course (Fig. 5).

**Fig. 6:**
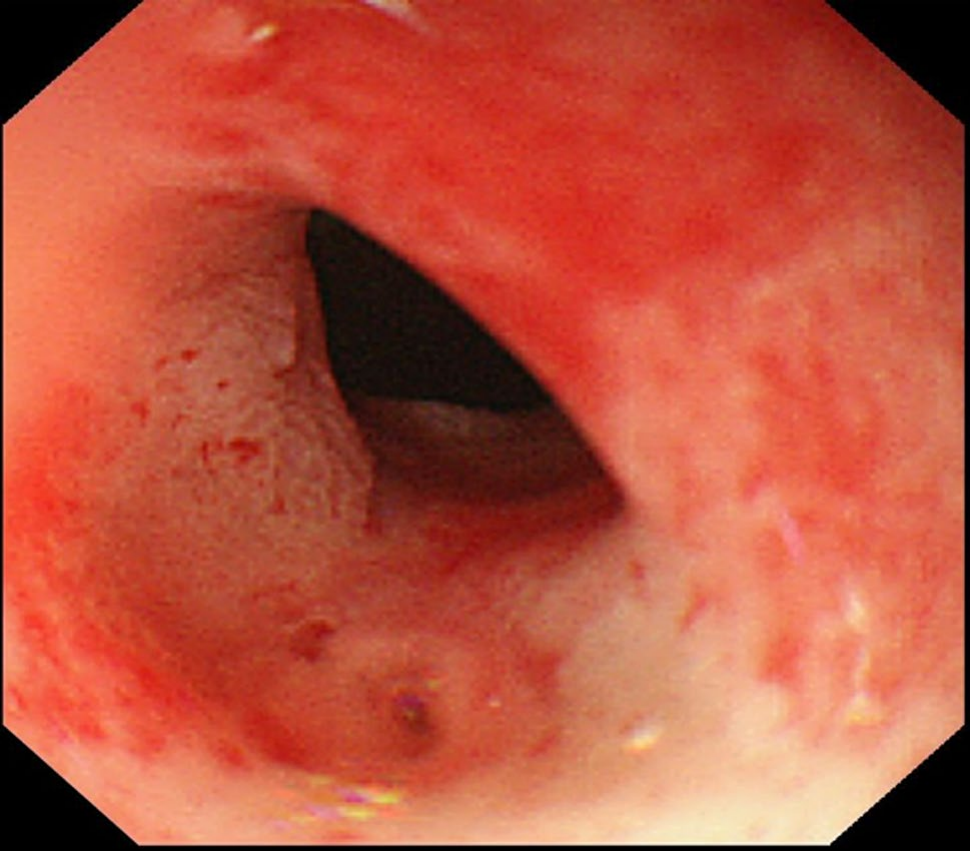
An upper gastrointestinal endoscopy performed 1 month after the completion of chemotherapy revealed the scarring of duodenal ulcerative lesion and the high-grade duodenal stenosis

One month after the completion of chemotherapy, nausea and vomiting after meals appeared. An upper gastrointestinal endoscopy revealed the scarring of the duodenal ulcerative lesion and the high-grade duodenal stenosis (Fig. 6); therefore, gastrojejunal anastomosis was performed. He has been well without recurrence for 4 years following the end of chemotherapy.

## Discussion

The incidence of pancreatitis by NHL involvement of the pancreas as the initial pathogenesis is quite rare [1, 3, 4, 7–9]; only 0.2–2% of patients with NHL have pancreatic involvement at the time of the first hospital visit [3]. A literature search of the PubMed database revealed some cases of primary pancreatic lymphoma presenting as acute pancreatitis [1, 7–9]. On the other hand, there have been only two cases of secondary pancreatic involvement causing acute pancreatitis, which were of peripancreatic lymphadenopathy and not duodenal lymphoma [3, 4]. The present report is the first to describe duodenal lymphoma as the cause of acute pancreatitis by involvement of the pancreas. This patient presented with typical abdominal pain and high levels of amylase and lipase. We excluded other etiologies of pancreatitis and, therefore, the involvement of duodenal lymphoma was indicated to be the main etiology of the observed acute pancreatitis, and R-CHOP therapy relieved the symptom of pancreatitis.

The present patient had been suffering from SLE for 15 years and developed NHL following tacrolimus treatment for 15 months for SLE. The risk of lymphoma in SLE is of considerable interest. It has been established that there are increased rates of malignancies in SLE: not only NHL, but also certain malignancies such as lung, hepatobiliary, vulvar/vaginal, and thyroid malignancies and cervical dysplasia [10]. Numerous cohort and case control studies have reported variable rates of risk of lymphoma among SLE patients. Some studies have reported that disease-related risk factors included long-term duration of SLE (12.4–17.8 years) at the time of NHL diagnosis [11, 12]. Bernatsky et al. [13] identified a higher risk of SLE-related lymphoma in men than in women, and this risk increased with older age, although these findings were of no particular significance. These findings, namely long disease duration and male sex, were relevant to the present case.

Conversely, studies have also demonstrated a relationship between exposure to medication and lymphoma development in SLE patients. A higher lymphoma risk in patients with exposure to cyclophosphamide and high cumulative steroids was seen in a multicenter SLE cohort analysis [14]. Alternatively, other studies have reported a negligible risk of treatment-associated lymphomas in SLE patients [15]. Therefore, the role of immunosuppressive drugs in SLE-related lymphoma does not seem to be crucial. As for tacrolimus, lymphoma development is extremely rare; Sekiguchi et al. [6] reported that the possibility of an association of oral tacrolimus with lymphoma without the history of transplantation has been suggested in only three cases. The primary diseases were mixed connective tissue disease, rheumatoid arthritis, and myasthenia gravis. The mean administration period before the diagnosis of lymphoma was approximately 18.7 months (10 months to 2 years). For treatment, chemotherapy was administered in two cases and radiotherapy in one case. Complete response (CR) was achieved in all three patients. In the present case, the period of tacrolimus administration was 15 months, and CR was achieved after R-CHOP was administered.

It was difficult to identify what was the risk factor for DLBCL, the SLE disease activity or the immunosuppressive therapy. In any case, it is important to take into consideration that both the long duration of the disease and immunosuppressive therapies carry the risk of lymphomas. In particular, the increased incidence of certain malignancies can be related to increased SLE disease activity as illustrated by the presence of inflammation. As gastrointestinal tract lymphomas have nonspecific symptoms, the diagnosis is often missed or delayed [16]. Thus, when inflammation findings are elevated or new lesions occur in SLE patients, a close imaging examination such as enhanced CT should be performed. In the present case, the symptom of pancreatitis enabled an early diagnosis of DLBCL. Acute pancreatitis can be a rare presentation of primary duodenal lymphoma, and appropriate evaluation can lead to earlier diagnosis and successful outcome, as seen in our case.
